# Individuals physically interacting in a group rapidly coordinate their movement by estimating the collective goal

**DOI:** 10.7554/eLife.41328

**Published:** 2019-02-12

**Authors:** Atsushi Takagi, Masaya Hirashima, Daichi Nozaki, Etienne Burdet

**Affiliations:** 1 Institute of Innovative ResearchTokyo Institute of TechnologyYokohamaJapan; 2 Imperial College LondonLondonUnited Kingdom; 3 Center for Information and Neural NetworksNational Institute of Information and Communications TechnologyOsakaJapan; 4 University of TokyoTokyoJapan; 5 Nanyang Technological UniversitySingaporeSingapore; University of Western OntarioCanada; University of California, BerkeleyUnited States

**Keywords:** motor control, human-human interaction, sensorimotor integration, computational neuroscience, haptic interaction, Human

## Abstract

How can a human collective coordinate, for example to move a banquet table, when each person is influenced by the inertia of others who may be inferior at the task? We hypothesized that large groups cannot coordinate through touch alone, accruing to a zero-sum scenario where individuals inferior at the task hinder superior ones. We tested this hypothesis by examining how dyads, triads and tetrads, whose right hands were physically coupled together, followed a common moving target. Surprisingly, superior individuals followed the target accurately even when coupled to an inferior group, and the interaction benefits increased with the group size. A computational model shows that these benefits arose as each individual uses their respective interaction force to infer the collective’s target and enhance their movement planning, which permitted coordination in seconds independent of the collective’s size. By estimating the collective’s movement goal, its individuals make physical interaction beneficial, swift and scalable.

## Introduction

A recent social experiment involving the widely acclaimed Pokemon video game ignited enormous public interest, where tens of thousands of players simultaneously controlled the protagonist of the game together and successfully finished the game (Zhang and Liu, 2015). Such collective behavior in humans has been researched when a collective makes a decision verbally (Webb, 1991; Hastie and Kameda, 2005; Bahrami et al., 2010). However, the key to many great human accomplishments, such as carrying stone blocks to construct the Great Pyramids, was enabled by many individuals who needed to coordinate the forces they applied on a stone in order to guide it on top of wooden rollers and move it. Such physical coordination has been investigated in pairs or dyads in the past decade (Basdogan et al., 2000; Sebanz et al., 2006; Reed and Peshkin, 2008; van der Wel et al., 2011; Malysz and Sirouspour, 2013).

Previous studies that investigated dyads found evidence of improved task performance (Basdogan et al., 2000; Reed and Peshkin, 2008; Malysz and Sirouspour, 2013), but the underlying mechanism of physical coordination was unknown. In a recent study, we tested dyads interacting in a continuous tracking task, and found that the tracking performance of both partners improved, even when the partner was worse at the task (Ganesh et al., 2014). This mutual improvement during continuous interaction is explained by a mechanism where individuals estimate the partner’s target from the interaction force to improve their prediction of the target’s motion (Takagi et al., 2017). In a second study, we showed that a stronger connection yields a better estimate of the partner’s target, enabling partners to improve more from the interaction (Takagi et al., 2018). We speak of the partner’s target for a tracking task, but this can be generalized to estimating a partner’s *movement goal*, which we define as the partner’s desired state, for example a position and velocity in time.

Although the mechanism of estimating the partner’s movement goal explains coordination in dyads, it is not known whether this interaction mechanism holds for an interactive tracking task with more than one partner. The connection dynamics to multiple partners may help inferior partners in a group but will likely hinder superior partners’ task performance. The dynamics may interfere with the coordination mechanism, which in dyads enabled even the superior partner to improve during the interactive tracking task (Ganesh et al., 2014). It is therefore unclear whether the interaction remains mutually beneficial for large groups.

To elucidate this question and investigate how collectives negotiate common actions, we examined a task inspired by dancing in which two, three and four partners have to control their motion while feeling forces from the soft interaction with others. We hypothesized that the stochastic summation of every partner’s actions, yielding the interaction force, would produce a noisier and poorer haptic estimate of the target as the group size increases. We also expected the connection dynamics and the collective’s inertia to have a detrimental effect on the superior partners’ performance. In such a scenario, the dynamics of being physically connected to a collective of partners may characterize the interaction behavior, similar to what was observed in joint reaching movements (Takagi et al., 2016). Could the coordination mechanism proposed in earlier studies (Takagi et al., 2017; Takagi et al., 2018) fully explain the tracking performance observed in collective interaction, or would the dynamics of the collective’s inertia outweigh the benefits of the coordination mechanism in larger groups?

## Results

**Figure 1.**
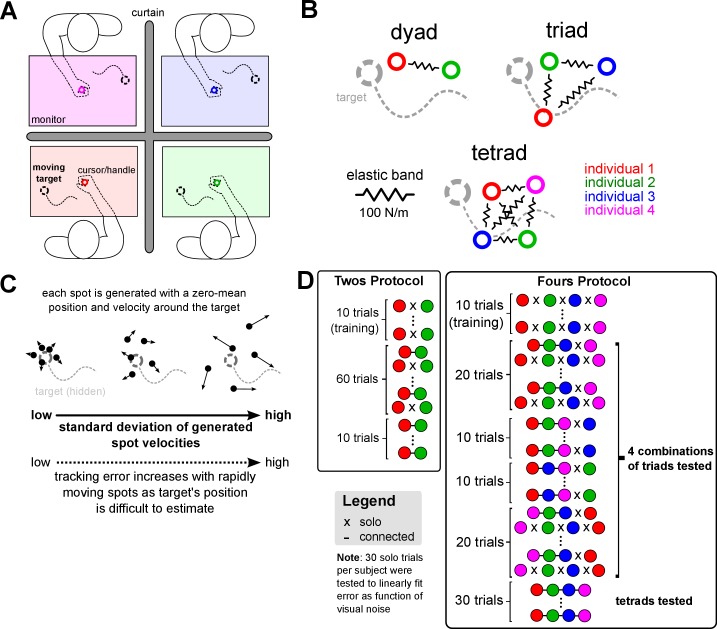
Dyads, triads and tetrads, whose right hands were connected, tracked a common target together to investigate collective physical interaction. (**A**) Subjects were recruited in twos or fours (schematic shows a tetrad). Each participant held onto a robotic handle with their dominant right hand to control a cursor on a monitor and track the same randomly moving target. Only one’s own cursor and the target were displayed on the monitor. (**B**) Physical coordination for dyads, triads and tetrads was enabled by forces exerted through the robotic handle that elastically connected all individuals’ right hands together. The interaction was removed in some trials to measure each individual’s solo tracking error as a function of the deviation in the target spots’ velocities. (**C**) The target was composed of five spots were spread thinly or widely to control each individual’s tracking performance. The deviation in the spots’ velocities, which was fixed during a trial, was randomized at every trial so participants could not know their skill relative to their partners beforehand. (**D**) Experimental protocol for twos and fours, where each circle represents an individual denoted by color. A dash indicates the individual was connected to partners. Both twos and fours experienced 10 solo training trials to become acquainted to the task. Twos then experienced 30 pairs of trials with and without the elastic connection, that is connected-solo-connected-solo etc. for 30 repetitions, and then 10 connected trials. For fours, three individuals of a tetrad were selected, forming all four different combinations of triads, and interacted in triads for one block per combination. In the first and last of these triad blocks, triads experienced 10 pairs of connected-solo trials, while the second and third triad blocks was composed of 10 connected trials. This was done to intersperse the solo trials to have a robust measure of the relationship between visual noise level and tracking error. In the final block, all individuals interacted as a tetrad for 30 trials.

We tested interaction in dyads, triads and tetrads who tracked a common target together using their right hands, which were all joined together with virtual elastic bands with a stiffness of 100 N/m (Figure 1A and B). 12 fours carried out the experiment in 12 triads and 12 tetrads, and 12 dyads were tested separately (see Figure 1D and the Materials and methods for details on the protocol). Individuals in the collective had to control a robotic handle using their right hand, which moved a cursor on their own respective monitor, to track a moving target (Figure 1A). The same target was used for all individuals of the collective. Each individual saw, on their own monitor, the positions of the target and their hand, but not the partners’ cursor positions. Individual performance at the task was calculated for each 15 s trial by measuring the average distance between their cursor and the target, defined as the *tracking error*. Two types of trials were tested: in *solo* trials, each individual tracked the target alone; in *connected* trials, the individuals’ right hands were coupled together by elastic bands.

**Figure 1—video 1.** The video shows a 15 second trial that each subject observed on their screen during training trials when the noise was lowest. The target is composed of five spots, which are regenerated every 400ms.

**Figure 1—video 2.** This video shows a 15-s trial where the visual noise was highest. Each of the five spots generated around the actual target position rapidly spread out as their velocity is picked from a wider Gaussian distribution.

**Figure 2.**
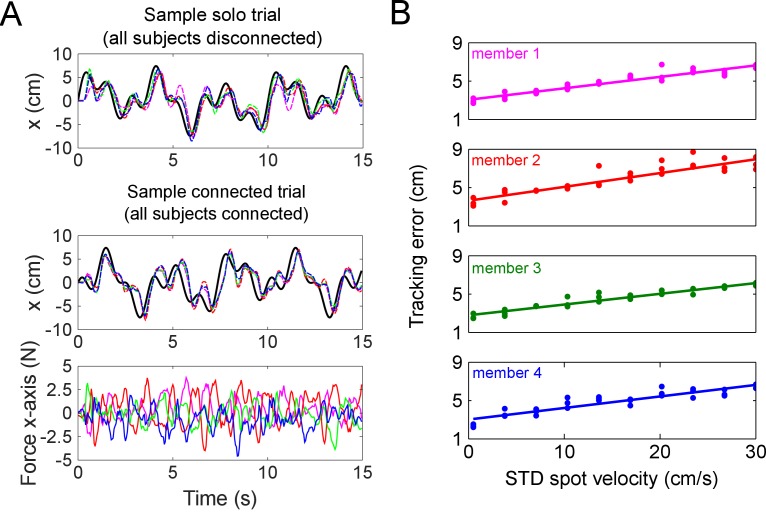
Sample trajectories from a single trial, and how the tracking error is tightly related with the visual noise imposed by the experimenter on each individual’s target. (**A**) Raw data showing the trajectories in the *x*-axis from a sample tetrad, where the black trace is the target and each dashed colored trace (red, green, blue and magenta) is one subject. The top panel shows the *x*-axis position of all subjects in a tetrad in a solo trial (all disconnected), whereas the bottom two panels are the *x*-axis position and force from a sample connected trial where all four subjects were coupled to each other via elastic bands. Subjects’ positions in both the sample solo and connected trials were all delayed with respect to the target due to visual feedback delays in anticipating the target’s motion. The force felt by each partner is approximately zero mean, and depended on each individual’s relative position to their partners in the group. (**B**) Linear fit of the standard deviation of spots’ velocities versus the tracking error from a sample tetrad. Each level of noise was tested for three trials without the elastic band to assess individual tracking error. This data was linearly regressed to estimate the expected tracking error of each individual as a function of the visual noise on the target imposed by the experimenter. This enabled us to test collectives composed of individuals with different tracking skill.

To test how interaction with inferior or superior partners influenced tracking performance, we *manipulated the tracking ability* of subjects by applying visual noise to the target (Körding and Wolpert, 2004) as described in the Materials and methods (see Figure 1C and Video 1 and 2 for visual noise during tracking task). The tracking error of subjects was linearly and tightly related to the standard deviation of this visual noise, such that greater visual noise resulted in larger tracking errors (see Figure 2B for sample subjects). A different amount of visual noise, which was randomly selected but fixed during each connected trial, was applied to each member of a collective for every trial. This enabled us to test the influence of interaction with participants of different selected tracking ability. As the visual noise was linearly related to the tracking error, we could calculate the change in each subject’s tracking error during the interaction relative to the visual noise that we applied. We dispersed the solo trials throughout the entire experiment to verify that the relationship between visual noise level and tracking error did not drift with time, for example due to fatigue, which is the rationale for having a complicated protocol for twos and fours (see Figure 1D).

Figure 2A shows raw data of the *x*-axis positions and forces experienced by a sample tetrad in solo and connected trials. The positions of the subjects lagged the target’s motion due to visual feedback delays in anticipating the target’s movement. For each subject, we assessed the *performance improvement *$1-e_{c}/e$, where $e_{c}$ was an individual’s tracking error in a connected trial and e was the same subject’s solo error, which was estimated from the visual noise applied during the connected trial. This ratio quantifies an individual’s tracking ability during the interaction relative to tracking alone. We analyzed this performance improvement as a function of the *partners’ relative error*, $1-e_{p}/e$, where $e_{p}$ was the mean of the partners’ solo errors, which was also estimated from the visual noise applied to the partners in the connected trial. This ratio is a measure of how the partners’ average tracking ability compared with the individual’s. This enabled us to study how each individual’s tracking ability changed when they interacted with ‘superior’ or ‘inferior’ partners.

**Figure 3.**
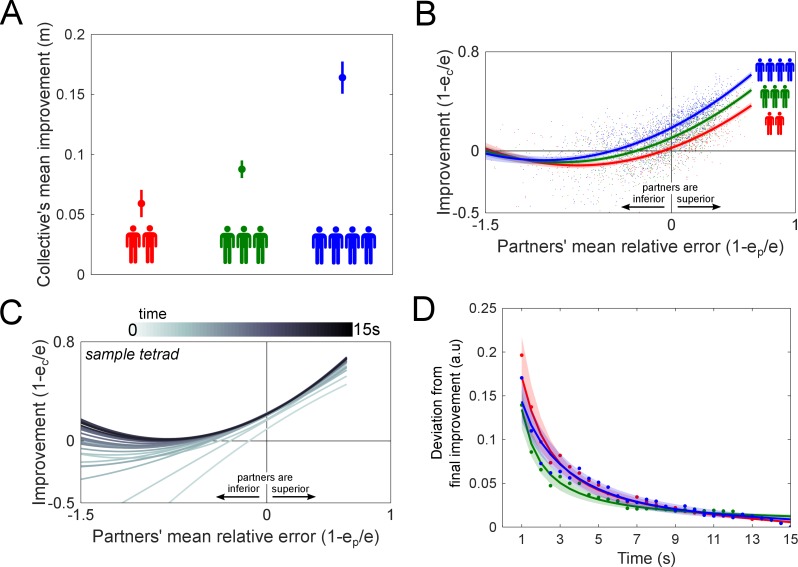
Collective physical interaction was surprisingly beneficial with coordination emerging rapidly in seconds, with the benefit in performance increasing with the number of partners. (**A**) The collective improvement for dyads, triads and tetrads increased with the collective’s size, reflecting the advantage of larger collectives. (**B**) Performance improvement as a function of the partners’ relative error for dyads (red trace), triads (in green) and tetrads (in blue). The solid traces come from a linear mixed-effects fit of the raw data (points come from all connected trials from all groups). Interacting with a superior group was found to improve one’s performance, which was graded by the collective’s size such that a larger collective resulted in more improvement. We expected a similar effect when interacting with an inferior collective, but interacting with more inferior partners did not degrade a superior member’s performance. (**C**) The performance improvement of a sample tetrad is plot in increments of 0.5 s from the start to the end of the trial as a function of the partners’ relative error. The improvement rapidly converged to the improvement curve observed in Figure 3B. (**D**) The deviation of the improvement curve in Figure 3B from the final improvement curve is plotted as a function of the trial time for dyads, triads and tetrads. The solid line is the mean of all groups, and the area represents one standard error. The rate at which they deviated from the collective mean was independent of group size. 10.7554/eLife.41328.007Figure 3—source data 1.Data of the partners’ mean relative error, improvement, group label and the group size used in the linear mixed-effects analysis.This data was also used in Figure 4 and 5.

The results of the collective physical interaction are plotted in Figure 3 (the data in Figure 3—source data 1 was used for all subsequent analysis). First, we assessed how the collective as a whole improved from the physical interaction, which is shown in Figure 3A, by taking the mean performance improvement from all individuals in the collective from every connected trial, and averaging over all trials for each collective. Two-sample t-tests revealed that the collective’s mean improvement increased with its size (between dyads and triads: *t*(22)=2.53, p<0.02; between dyads and tetrads: *t*(22)=6.07, p<10^−5^), revealing the benefits of interacting in larger collectives. To observe how each individual’s improvement changed as a function of the partners’ performance, we plotted each individual’s performance improvement as a function of the partners’ mean relative error for dyads (red trace), triads (green) and tetrads (blue) in Figure 3B. The data was fit using a linear mixed-effects model, where each recruited group of twos and fours were treated as a random factor to control for individual differences in their inherent ability to improve from the interaction (see Equation 2 in the Materials and methods for details). A mixed-effects analysis showed that the collective’s size modulated the individual’s performance improvement (χ^2^(2)=412, p<10^−15^, see Materials and methods for details).

We split the data into the *superior* ($1-e_{p}/e\;<\;0$) and *inferior* ($1-e_{p}/e\;>\;0$) *individuals* of the collective for dyads, triads and tetrads to examine how they were affected by physically interacting with superior or inferior partners. One-sample t-tests were carried out on the inferior and superior individuals’ improvements using a Bonferroni correction of significance 0.05/6. Inferior individuals improved when coupled to a superior collective, regardless of its size (dyads: *t*(11)=10.8, p<10^−6^; triads: *t*(11)=24.0, p<10^−10^, *t*(11)=23.0; tetrads: p<10^−9^). Surprisingly, superior individuals in dyads, triads and tetrads maintained their performance with respect to their solo error (dyads: *t*(11)=-2.22, p>0.05; triads: *t*(11)=-1.53, p>0.15; tetrads: *t*(11)=3.01, p>0.012). A superior individual could sustain their tracking performance even if they were physically coupled to an inferior collective regardless of how many inferior individuals were part of the collective.

An individual’s improvement was dependent on the performance of the others in the collective, but did the performance improvement change within the 15 s trial? We examined the improvement plot of Figure 3B for each collective as a function of time by calculating the improvement from the start of the trial to a specific trial time in increments of 0.5 s. Figure 3C shows the evolution of the improvement of a sample tetrad, where each trace is a second-order polynomial fitted to the data. The improvement was observed to significantly change over time. To study the evolution of the interaction’s beneficial effect on performance, we analyzed the improvement curve’s deviation from the *final improvement*, defined as the improvement at the end of the 15 s trial, that is the improvement during the entire trial, for dyads, triads and tetrads. Figure 3D shows the Euclidean distance between the second-order polynomial fits on the data at different times and the final improvement as a function of time for dyads, triads and tetrads. The improvement increased rapidly during the 15 trials for all collectives. To compare the rate of convergence between dyads, triads and tetrads, we fitted an exponential function to each collective of the form $a_{1}+a_{2}\text{exp}(-\lambda t)$, where $a_{1},a_{2}>0$ are parameters, λ>0 is the decay constant and t>0 is the trial time. Mann-Whitney U-tests revealed that the decay constant was similar between dyads and triads (*U*=122, n_1_=12, n_2_=12, p>0.11 two tailed), and between dyads and tetrads (*U*=136, n_1_=12, n_2_=12, p>0.44 two tailed). Thus, the time constant for the collective’s improvement did not depend on its size. Remarkably, it took only 7.4±0.9 s (mean ± standard error) for the collective to reach 90% of the final improvement.

**Figure 4.**
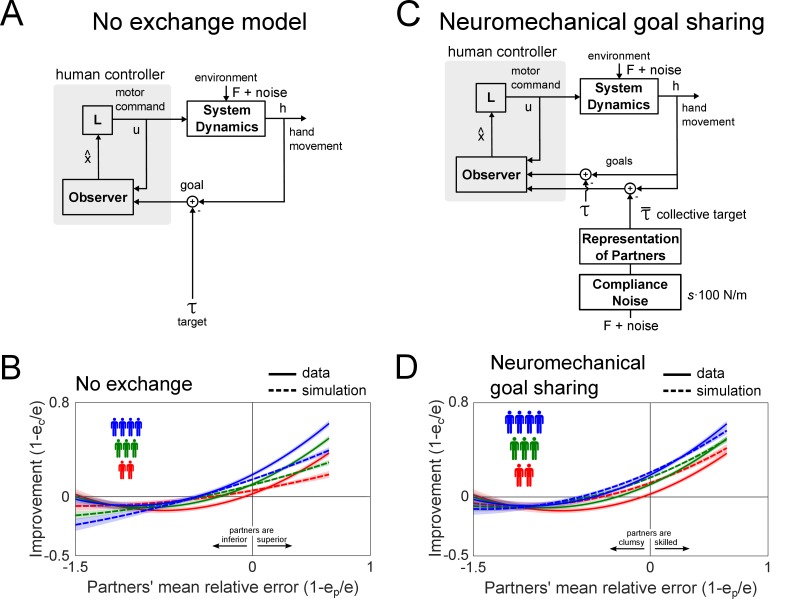
Simulations of the two proposed models of collective interaction and their predictions. (**A**) The *no exchange* model was simulated by assuming that individuals cannot interpret the interaction forces and track the target as if they were alone under the influence of the mechanics of the elastic band. (**B**) This model predicted a larger inferior collective to be a greater hindrance to a superior individual of the collective, unlike the data where superior individuals maintained their performance. (**C**) In the *neuromechanical goal sharing* model, each individual built a representation of the partners’ average behavior to estimate the collective target. (**D**) The predictions from this model best explained the improvement of both inferior and superior individuals in the collective, and its modulation with the collective’s size.

The empirical data shows that the collective physical interaction was beneficial for most individuals in the collective. How could individuals cause the performance improvement during collective interaction? To determine the behavioral strategy that individuals employed during collective interaction, we compared the empirical data from collective interaction with a simulation of it using the control models represented in Figure 4A and C to predict the outcome of the collective interaction experiment. In the simulation, we assumed that each individual sent motor commands to their arm to minimize the distance between their hand and the moving target. Simulated individuals relied on proprioception and vision for feedback of their hand and target positions, respectively. The simulated individuals had two free parameters that controlled the jerkiness of their movement and the strength of the controller, that is the control gain to bring the hand to the target. We carried out a sensitivity analysis to find values for these parameters that explained the empirical data best for each interaction model proposed in this study (see Supplementary material for details). Two, three and four such individuals were simulated in parallel with and without the elastic coupling to measure their performance at the tracking task during interaction and solo practice.

**Figure 4—figure supplement 1.**
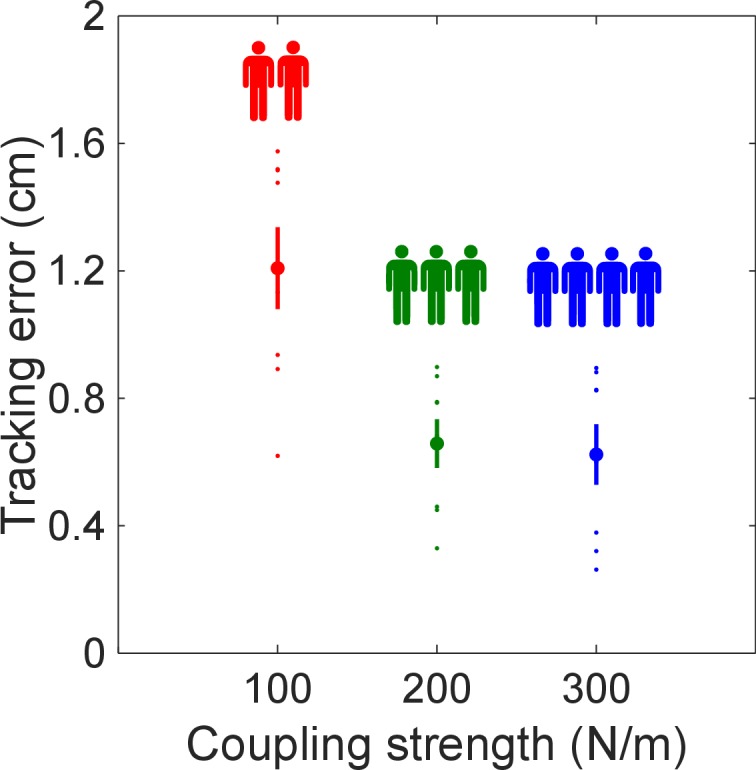
Results of the control experiment from a different set of subjects who tracked a haptic target without visual feedback. This experiment estimated how the interaction mechanics of coupling in dyads, triads and tetrads affected the quality of the estimated target from haptics. The compliance in the connection for dyads implies that dyads have more difficulty in estimating the collective target compared with triads and tetrads who felt a greater force. These values were used to adjust the *neuromechanical goal sharing* and the *source separation* limiting case.

**Figure 4—figure supplement 2.**
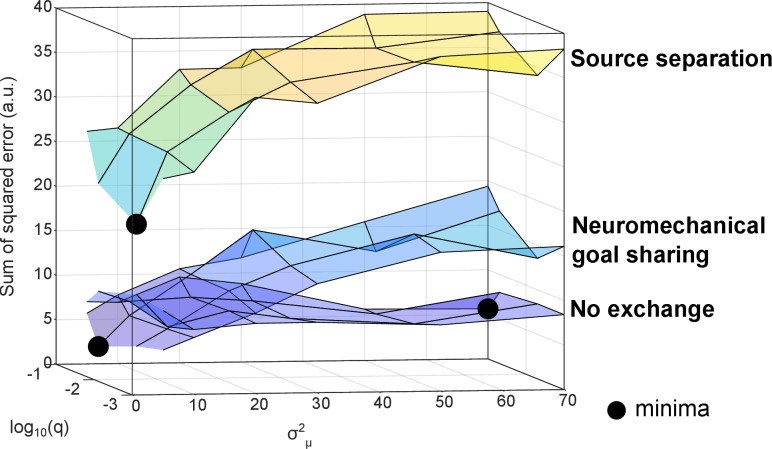
The sum of the sum of squared error between the fits from the data and the simulation as a function of q and $\sigma_{\mu }^{2}$. The SSE is relatively insensitive to changes in the strength q. The model with the lowest SSE is the *neuromechanical goal sharing* model.

We first tested whether the performance improvements observed in groups larger than dyads can be explained by a model where the physical connection to a superior or inferior collective with greater inertia dominates the interaction outcome. This model also tests whether the averaging of multiple partners’ trajectories during the tracking task helped to reduce tracking errors due to a cancellation of tracking errors. In this *no exchange* model (Figure 4A), individuals track a target estimated from vision whilst under the influence of the forces from the elastic bands. This model predicted an improvement that was linearly dependent on the partners’ relative error, which was different from the data (Figure 4B). Importantly, the model predicted that a superior individual in the collective was hindered by inferior partners, and the hindrance was greater with more inferior individuals in contrast to the data (Figure 3B). The mismatch between the experimental data and the *no exchange* model’s prediction for triads and tetrads suggests that individuals interacting in large groups use the interaction force to exchange information that is relevant to the task, as was found in dyads in our previous study (Takagi et al., 2017).

What kind of information did the individuals in triads and tetrads estimate from the interaction with their partners during the tracking task? In earlier studies (Takagi et al., 2017; Takagi et al., 2018), we showed that partners in dyads estimated each other’s target through the interaction force to improve their prediction of the target’s motion. Individuals in triads and tetrads may also extract useful information from haptics to improve tracking performance. We hypothesized that individuals interpret the summed interaction force as originating from one entity that tracks a *collective target*. According to this hypothesis, the individuals’ central nervous system (CNS) recognizes some correlation between the interaction force and the target motion (Parise and Ernst, 2016), then builds a representation of the entity that tracks the target. We assume that every individual’s CNS in the group estimates one collective target from the summed interaction force regardless of the number of partners in the group. In this extended *neuromechanical goal sharing* model, we propose that individuals track the optimally weighted average of the collective target and one’s own target from vision (see Figure 4C for schematic of the model).

As an example, for tetrads, we simulated four connected individuals who each estimated a collective target from the three other partners, and who then integrated this haptic estimate of the target with their own visual estimate of the target’s position (see Equation 9 in the Materials and methods). The weights between vision and haptics were assumed to be known by every partner as we were interested in comparing the steady-state predictions of the model with the data. Furthermore, we accounted for the additional haptic noise that arises due to the compliance of the spring connection. In our earlier study (Takagi et al., 2018), we found that a stiffer spring reduces the haptic noise when estimating the partner’s target. Mechanics tells us that an individual in a triad who is connected to two partners by a total of two springs, each of stiffness 100 N/m, feels an equivalent force to being connected to the average of the two partners’ positions by a spring of stiffness 200 N/m (see Equation 6 in Materials and methods) (Burdet et al., 2013). In other words, individuals in larger groups effectively feel like they are connected to the group’s average position by a stronger spring. The *error due to a specific compliant connection* was accounted for in the simulations as additive noise (see Materials and methods for details on the haptic tracking experiment to measure this additional error due to the compliance in the spring in dyads, triads and tetrads, and Figure 4—figure supplement 1 for the results of the haptic tracking experiment).

The simulation of the *neuromechanical goal sharing* model predicted a performance improvement that captured the curvature of the improvement as a function of the partners’ relative error with minimal deviation from the data when tested in a sensitivity analysis (Figure 4D and Figure 4—figure supplement 2). The performance improvement increased supralinearly for inferior individuals, and superior individuals retained their performance even when coupled to an inferior collective. Furthermore, the improvement was correctly modulated by the collective’s size, such that tetrads improved the most, followed by triads, and then dyads. These results suggest that individuals in collectives of different sizes use the same coordination strategy of extracting a haptic estimate of the collective target position from the interaction force.

## Discussion

This study tested physically interacting dyads, triads and tetrads in a tracking task to assess the effect of the group’s size and its skill on the participating individuals’ tracking performance. We found that the total group’s performance increased with the group size, where inferior individuals in the group improved incrementally more in larger groups, and superior individuals were capable of sustaining their superior tracking performance even when connected to a group of individuals with inferior performance. Contrary to the results of our previous study (Ganesh et al., 2014), the superior individuals of the dyads in the current study did not improve. This discrepancy in the results is likely due to the high amount of visual noise added to the target in order to manipulate each individual’s tracking performance.

In our experiment, the performance improvements observed in dyads, triads and tetrads did not arise instantaneously, but emerged continuously during the trial such that 90% of the group’s final performance improvement (calculated over the entire trial) was reached after 7 s. As Figure 3C shows, the partners’ movements initially depend only on the connecting spring dynamics (compare with Figure 4A and B), and gradually acquire a model of the interaction dynamics enabling them to benefit from this interaction. The similarity in the adaptation rates between dyads, triads and tetrads in reaching their performance improvement at the end of the trial may indicate that the same coordination mechanism may be utilized regardless of the size of the interacting group. The similarity in these adaptation rates for physical interaction stands in contrast with verbal or gestural communication where significantly longer time is needed with more participants. This highlights the advantage of the simultaneity of haptic communication relative to the sequential exchanges in verbal and gestural communication.

In order to identify the coordination mechanism that explained the improvements from collective physical interaction, we used a computational model to test the determinants of interaction, to predict their effect on the performance improvement, and compare the predictions with the empirical data. The *neuromechanical goal sharing* model, which captured the improvements from the empirical data, suggests a mechanism whereby individuals extract task relevant sensory information from haptics, and integrate it with their own visual information of the target’s motion to improve tracking performance during interaction. In this model, we assumed that each individual extracts a haptic estimate of the target from the interaction force. As this haptic estimate of the target is stochastically optimally combined with the individual’s visual target, this improves their tracking performance even when connected to partners having a collectively inferior performance. The haptic estimate of the target arises from the summed interaction force, which is composed of the elastic couplings to multiple partners, that is equivalent to one elastic coupling to the average partner (see Equation 6 in the Materials and methods).

**Figure 5.**
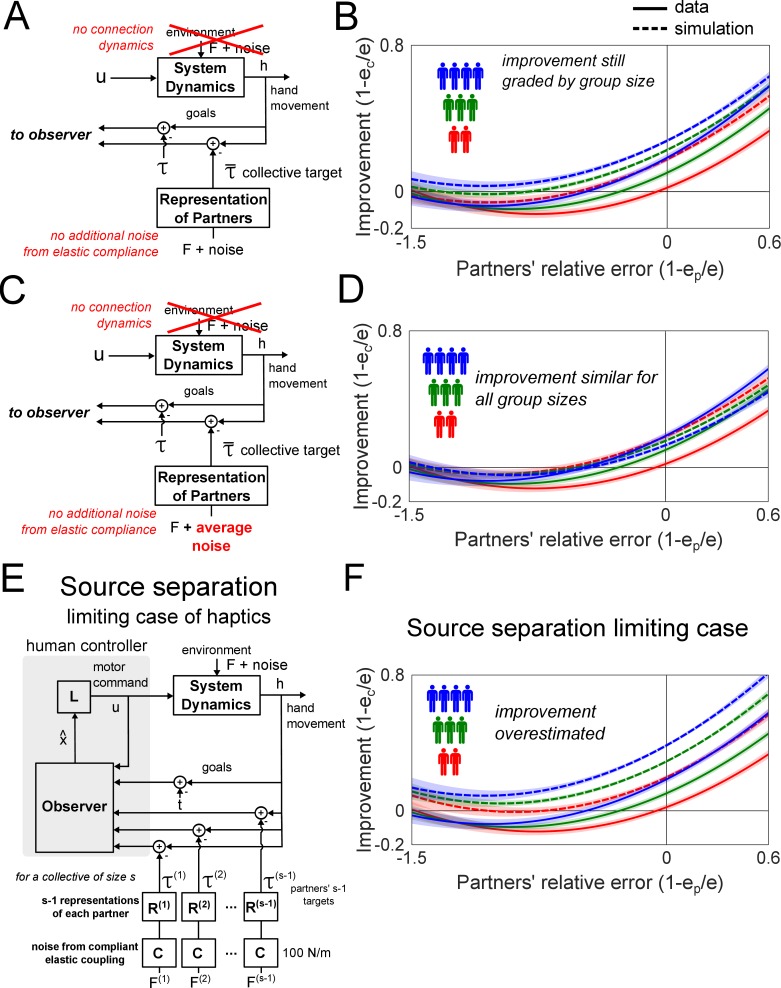
Simulation to examine the mechanism underlying the graded improvement and the limits of sharing information through haptics. (**A**) A schematic of the *neuromechanical goal sharing* model without the dynamics of the spring and without the additional haptic noise due to the compliance of the spring. The simulation of this model are shown in (**B**), where the improvements remained graded by group size, implying that the performance was graded for a different reason. (**C**) The *neuromechanical goal sharing* model was simulated without the dynamics of the spring, without the additional haptic noise due to the compliance of the spring, and assuming that the noise in the interaction force was the average partners’ visual tracking noise. The simulation of this model is shown in (**D**). Since the noise was averaged, the group size had no effect on the improvement, which was similar for dyads, triads and tetrads. Thus, the graded improvement due to group size arose from the stochastic summation in the interaction force. (**E**) The *source separation* limiting case assumes that each individual receives additional sensory information of the target position through the interaction force from every partner. (**F**) The *source separation* case significantly overestimates the improvement for dyads, triads and tetrads, indicating that the individuals could not separate the sources of the interaction force.

If the haptic estimate of the target is extracted from summed interaction force, which is a function of the average partner’s movements, then intuitively the performance improvement from integrating this haptic signal should depend only on the average partner’s tracking error, and not on the number of partners in the collective. If so, why did the simulation in Figure 4D of the *neuromechanical goal sharing* model predict improvements that were dependent on both the average partner’s error and the size of the collective? There may be two main reasons for the graded performance improvement with group size. First, the connection dynamics alone could have graded the improvement, since the *no exchange* model (in Figure 4A and B) also predicted improvements that were graded by group size. Second, the effect of the additional noise in the haptic estimate of the target due to the compliance of the elastic coupling may explain the graded improvement (see Equation 16 in Materials and methods). To assess the impact of these two factors on the predicted performance improvement, we simulated the *neuromechanical goal sharing* model (see Figure 5A) without the interaction spring dynamics (F=0 in Equation 6 in the Materials and methods) and without the additional noise from the elastic compliance (${\left(\sigma_{c}^{\left(i\right)}\right)}^{2}=0$ in Equation 16 in the Materials and methods). As the results still exhibit improvements graded as a function of group size (see Figure 5B), the graded improvement was not caused by the connection dynamics nor by the additional haptic noise due to the elastic compliance.

What explains the grading of the improvement as a function of both the average partner’s error and the collective’s size? The original intuitive premise must be questioned as to whether the improvement from interacting with a collective of partners, whose mean tracking error is $\bar{e}$, is the same as the improvement from interacting with one average partner who has the error $\bar{e}$. In previous studies (Takagi et al., 2017; Takagi et al., 2018), the noise in the haptic measurement of the target was equivalent to the partner’s visual tracking noise. So what is the noise in haptics from an interaction force during collective interaction? Although the interaction force is equivalent to one stiffer elastic coupling to the average partner (as Equation 6 shows), the noise in this interaction force is not the average of the partners’ visual tracking noise. Instead, the stochastically summed interaction force has noise that is inversely proportional to the squared number of partners (see Equation 11 in Materials and methods), which is different from the mean of the partners’ visual tracking noise. To illustrate this difference, we simulated the *neuromechanical goal sharing* model where the haptic noise was the mean of the partners’ visual tracking noise (see Figure 5C). To isolate the effects of the haptic measurement noise, we again removed the connection dynamics and the noise from the elastic coupling in this simulation. This model predicted similar improvements irrespective of group size (see Figure 5A), showing that the graded improvement as a function of group size is indeed explained by a reduction in the variance of haptics due to the averaging of the partners’ positions in the interaction force.

Does the elucidated mechanism provide maximum possible performance improvement with haptic feedback? Maximum information transfer during collective interaction can be estimated in a limiting case of the *neuromechanical goal sharing* model where the central nervous system is able to extract every partner’s contribution to the interaction force (instead of modeling the interaction force as coming from a single entity). This would be similar to the cocktail party effect of audition where one can isolate a conversation in a room of people talking at the same time (Hawley et al., 2004). Each individual might extract multiple streams of information from the interaction force (one, two and three streams for dyads, triads and tetrads, respectively) using individual spectral characteristics, yielding the maximal possible information transfer through haptics (see Figure 5E for the schematic of this model). However, the predictions of this *source separation* limiting case (see Figure 5F) consistently overestimated the improvement in comparison to the data, showing that our individuals could not break down each individual source in the interaction force. Instead, the average behavior of all other individuals was identified, and their *collective target* was inferred. This reveals a limit in the ability to share and estimate information through haptics.

In summary, this paper presented experiments and computational modeling to understand how physically interacting human individuals coordinate their movements during the collective tracking of a common target. The results elucidate the coordination mechanism in a collective by systematically analyzing how the information from the interaction dynamics is processed by its individuals. As the interaction force is the sum of all partner’s forces, it is not possible to identify each partner’s specific contribution to it. Instead, the individuals estimate *a collective target* from the interaction force, which they combine with their own visual target. The performance improvement resulting from this mechanism is suboptimal relative to that allowed by a putative source separation mechanism, but it still enables the collective’s individuals to improve their tracking error when interacting with superior partners, and to not be hindered by inferior ones. This neuromechanical coordination mechanism is also scalable, as the time required to adapt to the group’s skill is independent of group size, and a group’s total performance improvement increases with its size. The surprising result that the collective’s mean improvement increases in larger groups is explained by the stochastic properties of the collective target that is extracted from the summed interaction force.

## Materials and methods

### Experiments

The study was conducted according to the Declaration of Helsinki and was approved by the ethics committee of the Graduate School of Education at the University of Tokyo (reference number 14-75). Each of the 72 subjects gave a written consent prior to starting with the experiments. The sample size of 12 per group of dyads, triads and tetrads was determined by a prior power analysis from a repeated-measures ANOVA within and between interaction consisting of 3 groups and the error detection parameters α=0.05 and β=0.8 with a medium effect size of $\eta^{2}=0.06$.

Each subject held onto the robotic handle of the Phantom 1.5HF (Geomagic), which constrained the handle’s movement within a horizontal plane via software. The individual monitors displayed a cursor of the handle position and the target, which was composed of a dynamic cloud around the multi-sine function

(1) $$\begin{array}{c} x\left(t\right)=1.6\sin\left(0.1t\right)+4\sin\left(0.3t\right)+0.8\sin\left(0.5t\right)+2.4\sin\left(0.8t\right)\\ y\left(t\right)=0.8\sin\left(0.2t\right)+2.4\sin\left(0.3t\right)+4\sin\left(0.6t\right)+2.4\sin\left(0.8t\right) \end{array}$$

where the target’s trajectory was randomized through the selection of the initial time according to a uniform stochastic distribution in the interval between 0 and 10 s.

The dynamic cloud consisted of five circular spots that were displayed every millisecond (as shown in the Video 1 and 2). Each spot was regenerated one at a time every 400 ms by picking a new position and velocity. These position and velocity parameters were determined at the start of a trial from normal random distributions with a standard deviation of 0.005 m for the position, and from a set of ten equally spaced values from 0.005 m/s to 0.3 m/s for the velocity. The wider the spots were spread, the more difficult it was to follow the target as its true position was hard to guess (Körding and Wolpert, 2004). Spots with low velocity noise were easy to track but high velocity noise spots spread out rapidly like fireworks. Every time a velocity noise was selected for each subject, it was removed from the set that was unique to each subject. The random selection ensured that an individual’s own performance and the others’ tracking skill were unknown a priori.

The velocity parameter enabled us to control the *tracking error* of each individual in a trial, which was measured as the root-mean squared distance between the target and the cursor. For each subject, the tracking error on trials without interaction was regressed with the target spot velocity noise using data from three trials per velocity noise level, giving a fit with R^2^ = 0.80 ± 0.01 (mean ±standard error for all subjects). The spot velocity noise was used as an estimate of each individual’s tracking error (see Figure 2B for fits from a sample tetrad).

Subjects were instructed to follow the target as accurately as possible and were told that they would experience forces on their hand. At the end of the experiment, subjects were asked about the nature of the forces. Although some guessed that the forces originated from a partner, none of them could tell how many partners they were connected to.

The experimental protocol is described in Figure 1D. Twos experienced 80 total trials and fours completed 100 trials in total. Both twos and fours experienced 10 solo training trials to become acquainted to the task. After this training phase, twos and fours encountered a series of solo and connected trials. Twos then carried out 60 trials with and without the elastic connection in a series of 30 connected-solo trials, and then 10 connected trials. This ensured that solo trials were interspersed throughout the experiment. Interaction data from both triads and tetrads were collected during the experiment with fours. We collected as much data as possible from triads by testing all four combinations of triads possible from the tetrad, and collected the tetrad interaction data in the last 30 trials. Thirty solo trials were interspersed such that 10 were tested after training, 10 prior to tetrad interaction, and another 10 in each triad block where the excluded individual experienced solo trials instead of triad interaction trials. In total, 40 connected trials for dyads and triads, and 30 connected trials for tetrads.

### Analysis

A linear mixed-effects model

(2) $$\Delta c=\beta_{0\rho }+\beta_{1\rho }s+\beta_{2\rho }\Delta p+\beta_{3\rho }\Delta p^{2}+\beta_{4\rho }\Delta p^{3}+\beta_{5\rho }\left(\Delta p\cdot s\right)+\varepsilon_{\rho }$$

was employed to fit the improvement $\Delta c=1-e_{c}/e$, where e is the error of an individual in a solo trial (estimated from the linear regression with the visual noise) and $e_{c}$ is the same subject’s error on a connected trial, as a function of the partners’ relative error, $\Delta p=1-e_{p}/e$, where $e_{p}$ was the partners’ mean error estimated from solo trials, and the collective’s size s. In this model, $\beta_{0\rho }$ is the intercept, $\beta_{1\rho }$ to $\beta_{5\rho }$ are the parameters for each predictor and $\varepsilon_{\rho }$ is the unexplained variance of the improvement for each collective ρ.

### Simulation model

A model was developed in discrete time $\left\{kdt,k=1,2,\ldots \right\}$ to simulate how the members of a collective connected by elastic bands plan their movement to track a randomly moving target in two dimensions. The Cartesian product of two one-dimensional models as described below was used in simulation. At every time index the target with position $\tau_{k}$ must be estimated, then a motor command $u_{k}$ is generated to move the hand’s position $h_{k}$ to the target. First, we describe the state equation that governs the movement of the target, and then that of the hand, and combine these two equations to formulate a single state equation of the full system. The movement of the target, which is assumed to be moving randomly via Gaussian noise in its velocity ${\dot{\tau }}_{k}\equiv \mu_{k}$, is described by the first-order system

(3) $$\tau_{k+1}={A\tau }_{k}+\left[\begin{array}{c} 0\\ dt \end{array}\right]\mu_{k}\;,\;\;A\equiv \left[\begin{array}{cc} 1 & dt\\ 0 & 1 \end{array}\right],\;\;\mu_{k}\in ({0,M}_{k})$$

where $\tau_{k}\equiv {\left[\tau_{k},{\dot{\tau }}_{k}\right]}^{T}$ is the *target state* and $M_{k}\equiv E\left[\tau_{k}\;\tau_{k}^{T}\right]$ is the covariance matrix.

The control of the hand is modelled as $m{\ddot{h}}_{k}=u_{k}+F_{k}$ with point-mass m and the force F from one or several elastic bands. In state-space format, this yields

(4) $$h_{k+1}=Ah_{k}+B\left(u_{k}+F_{k}\right)\;,\;\;h_{k}\equiv {\left[h_{k},{\dot{h}}_{k}\right]}^{T},\;\;B\equiv \left[\begin{array}{c} 0\\ dt/m \end{array}\right]$$

where the control command *u* to move the hand towards the target is described by

(5) $$u_{k}=-\left[L_{p},L_{v}\right]\left(h_{k}-\tau_{k}\right)$$

with $L_{p}$ and $L_{v}$ describing the position and velocity control gains, respectively. In a collective of individuals $\left\{i=1\ldots s\right\}$, each individual’s right hand with state $h_{k}^{\left(i\right)}\equiv {\left[h_{k}^{\left(i\right)},{\dot{h}}_{k}^{\left(i\right)}\right]}^{T}$ is connected to the s-1 other individuals’ right hands through elastics bands of stiffness K>0 and damping D>0 that produce the force

(6) $$F_{k}^{\left(i\right)}=\sum_{j\neq i}K\left(h_{k}^{\left(j\right)}-h_{k}^{\left(i\right)}\right)+D\left({\dot{h}}_{k}^{\left(j\right)}-{\dot{h}}_{k}^{\left(i\right)}\right)\;=\;\left(s-1\right)K\left({\bar{h}}_{k}^{\left(i\right)}-h_{k}^{\left(i\right)}\right)+\left(s-1\right)D\left({\dot{\bar{h}}}_{k}^{\left(i\right)}-{\dot{h}}_{k}^{\left(i\right)}\right)$$

where ${\bar{h}}_{k}^{\left(i\right)}=\frac{1}{s-1}\sum_{j\neq i}h_{k}^{\left(j\right)}$ is the average partners’ position. Importantly, as we see in the right side of this equation, the sum of the elastic coupling to all partners is equivalent to the interaction force when coupled with a more rigid and damped elastic interaction with the average of the partners’ hands. Solo trials, where the subjects are not connected, are characterized by zero force $F_{k}^{\left(i\right)}\equiv 0$ for all i. A subject using the motor command of Equation 5 to move their hand according to Equation 4 to follow the target, with motion described by Equation 3, is described by the full state equation

(7) $$x_{k+1}=Ax_{k}+B\left(u_{k}+F_{k}+m\mu_{k}\right)\;,\;\;x_{k}\equiv h_{k}-\tau_{k}$$

which is equivalent to the difference of Equation 4 minus Equation 3.

### Models of interaction

Two models of interaction are described from the sensory information exchange between the partners. First, we describe the solo strategy of one subject tracking the target $\tau_{k}$ alone using only visual feedback. To generate the motor command according to Equation 5, the state describing the difference between the target and the hand is observed through

(8) $$z_{k}=h_{k}-\tau_{k}+\nu_{k}$$

where the observation $z_{k}$ is corrupted by Gaussian visual noise $\nu_{k}$ with variance $\sigma^{2}\equiv E\left[{\left(z_{k}-E\left[z_{k}\right]\right)}^{2}\right]$. The linear quadratic estimation is computed in discrete time using an iterative Kalman filter algorithm (Kalman, 1960). Sensory delay in vision and proprioception is compensated for by integrating Equation 7.

Now that we have described how to visually track a target, what motion planning model could be used to track the randomly moving target whilst being physically coupled to multiple partners? In the *no exchange* model, each individual ignores the interaction forces and tracks the target using the visual information of the target’s position, as in Equation 8, under the influence of the dynamics of the elastic bands described in Equation 6. The *neuromechanical goal sharing* model (Takagi et al., 2017; Takagi et al., 2018) proposes that, in dyads, both individuals extract a haptic estimate of the target’s position from the interaction force with the partner, and optimally combine it with their own visual estimate of the target. Similarly, we propose that in collective interaction each individual i uses the interaction force to extract a haptic estimate of the target position, referred from here on as the *collective target *${\bar{\tau }}_{k}^{\left(i\right)}$, such that the observation of the difference between the hand of individual *i* and the target is observed using

(9) $$z_{k}^{\left(i\right)}=\left[\begin{array}{c} h_{k}^{\left(i\right)}-\tau_{k}^{\left(i\right)}\\ h_{k}^{\left(i\right)}-{\bar{\tau }}_{k}^{\left(i\right)} \end{array}\right]$$

which extends the corresponding law of previous studies (Takagi et al., 2017; Takagi et al., 2018).

What is the variance of the noise that corrupts the haptic measurement of the collective target in the extended *neuromechanical goal sharing* model? In previous studies (Takagi et al., 2017, Takagi et al., 2018), the interaction; force was linearly dependent on the partner’s hand position, and so the noise in the haptic measurement of the partner’s target was the partner’s visual tracking noise. Similarly, in collective interaction, the collective target is estimated from the interaction noise, which is shown in Equation 6 to be linearly dependent on the partners’ average hand position ${\bar{h}}_{k}^{\left(i\right)}$. Let every j^th^ partner’s visual measurement of the target be corrupted by Gaussian visual tracking noise with variance ${\left(\sigma^{\left(j\right)}\right)}^{2}$. Then the difference between the hand and the collective target’s position $h_{k}^{\left(i\right)}-{\bar{\tau }}_{k}^{\left(i\right)}$ suffers Gaussian noise with variance

(10) $${\left({\bar{\sigma }}^{\left(i\right)}\right)}^{2}=\frac{1}{{\left(s-1\right)}^{2}}\sum_{m,n\neq i}cov\left(m,n\right)\;.$$

We can assume that these measurements between partners are independent, thus $cov\left(m,n\right)=0\forall m\neq n$ and $cov\left(n,n\right)={{(\sigma }^{\left(n\right)})}^{2}$, and the variance in the measurement of the collective target

(11) $${\left({\bar{\sigma }}^{\left(i\right)}\right)}^{2}=\frac{1}{{\left(s-1\right)}^{2}}\sum_{j\neq i}{\left({\bar{\sigma }}^{\left(j\right)}\right)}^{2}$$

is inversely proportional to the number of partners in the collective. Therefore, the measurement noise on the collective target will reduce in larger collectives even if the partners’ average tracking noise is equivalent.

We further tested a modified version of the *neuromechanical goal sharing* model (see Figure 5C and 5D) with the intuitive, but incorrect, expectation that the interaction with multiple partners whose average error is ${\bar{e}}^{\left(i\right)}$ is identical to interacting with one partner whose error is equivalent to ${\bar{e}}^{\left(i\right)}$. In this scenario, the variance of the noise in the haptic measurement of the collective target would be equal to the average of the partners’ visual tracking noise, that is ${\left({\bar{\sigma }}^{\left(i\right)}\right)}^{2}=\frac{1}{s-1}\sum_{j\neq i}{\left({\bar{\sigma }}^{\left(j\right)}\right)}^{2}$, with a denominator different from Equation 11. If this were the noise in the haptic estimate of the collective target, the improvement would not change with the group’s size (as Figure 5D shows), which is in contrast to what is observed in the data.

How does an individual *i* estimate the collective target of Equation 9 from the interaction force in Equation 6? The average of the partners would use a motor command similar to Equation 5,

(12) $${\bar{u}}_{k}^{\left(i\right)}=-\left[{\bar{L}}_{p}^{\left(i\right)},{\bar{L}}_{v}^{\left(i\right)}\right]\left({\bar{h}}_{k}^{\left(i\right)}-{\bar{\tau }}_{k}^{\left(i\right)}\right)\equiv -{\bar{L}}^{\left(i\right)}{\bar{x}}^{\left(i\right)}$$

where the average partner’s state is estimated through the force and the state of one’s own hand, whilst the average partner’s control law ${\bar{L}}^{\left(i\right)}$ from Equation 12 is identified by letting it evolve with noise according to

(13) $${\dot{\bar{L}}}^{\left(i\right)}\equiv \lambda \;,\;\;\;\lambda \in N\left(0,E\left[{\left({\dot{\bar{L}}}^{\left(i\right)}\right)}^{T}{\dot{\bar{L}}}^{\left(i\right)}\right]\right)\;.\;$$

Thus, the representation of the partner includes the state of their hand, their target, their control law, one’s own hand and the elastic force to yield

(14) $$\xi \equiv \left({\bar{\tau }}^{\left(i\right)},\;{\bar{h}}^{\left(i\right)},\;{\bar{L}}^{\left(i\right)},\;h^{\left(i\right)},\;F^{\left(i\right)}\right)\;.$$

This state ξ is described by the non-linear function $\dot{\xi }\equiv f\left(\xi \right)$ that is linearized at every time step to be used for linear quadratic estimation (Ljung, 1979). ${\bar{L}}^{\left(i\right)}$ is identified by minimizing the squared estimation error of the observations of one’s own target position $\tau_{k}^{\left(i\right)}$, hand position $h_{k}^{\left(i\right)}$ and force $F_{k}^{\left(i\right)}$. Once ${\bar{L}}^{\left(i\right)}$ is identified, the collective target ${\bar{\tau }}^{\left(i\right)}$ is estimated by minimizing the squared estimation error of the observations of the average partner’s estimated control ${\bar{L}}^{\left(i\right)}$, one’s own hand position $h_{k}^{\left(i\right)}$ and the force $F_{k}^{\left(i\right)}$.

The *source separation* limiting case is where every partner’s target can be estimated and combined with one’s own visual estimate of the target,

(15) $$z_{k}^{\left(i\right)}=\left[\begin{array}{c} h_{k}^{\left(i\right)}-\tau_{k}^{\left(1\right)}\\ \vdots \\ h_{k}^{\left(i\right)}-\tau_{k}^{\left(s\right)} \end{array}\right]\;.$$

In this limiting case, s−1 observations of the partners’ target position are directly provided to each individual in the collective, who integrates the partners’ targets with their own visual estimate of the target, providing s total observations of the target.

### How compliance changes the quality of haptic information

In a previous study (Takagi et al., 2018), we found that the strength of the elastic coupling influenced the quality of the haptic information. With a weaker elastic band, the amplitude of the interaction force is smaller, reducing the signal-to-noise ratio when measuring it through haptics. Dyads, triads and tetrads experienced different magnitudes of force due to the increasing number of elastic bands that coupled them together. The dynamics experienced by dyads, triads and tetrads can be modeled as a single elastic band of 100 N/m, 200 N/m and 300 N/m respectively, which connects each individual to the average position of the partners as shown in Equation 6.

Another eight subjects were recruited individually to carry out a *haptic tracking control experiment*. The target movement was the same as in Equation 1, but without visual feedback, that is the target was invisible to the subject. Subjects tracked the haptic target for 15 s, and experienced five trials of each coupling stiffness consecutively in the order of 300 N/m, 200 N/m and 100 N/m, respectively. Figure 4—figure supplement 1 shows the results of this experiment, revealing that stronger stiffness resulted in lower tracking errors. The values found in this experiment were used to alter the *source separation* and *neuromechanical goal sharing* models by changing the sensory noise in the haptic estimate of the collective target. The haptic noise from Equation 11 has some additive noise ${\left(\sigma_{c}^{\left(i\right)}\right)}^{2}$ due to the compliance of the elastic connection,

(16) $${\left(\sigma_{h}^{\left(i\right)}\right)}^{2}={\left({\bar{\sigma }}^{\left(i\right)}\right)}^{2}+{\left(\sigma_{c}^{\left(i\right)}\right)}^{2}\;.$$

In the haptic tracking experiment, we measured the additional error in the tracking task that arises due to the softness of the elastic spring. These error values can be used to estimate ${\left(\sigma_{c}^{\left(i\right)}\right)}^{2}$, and so the haptic noise is described by

(17) $${\left(\sigma_{h}^{\left(i\right)}\right)}^{2}={\left({\bar{\sigma }}^{\left(i\right)}\right)}^{2}+\;\psi \left(\sigma_{\mu }^{2},q,{\left({\bar{\sigma }}^{\left(i\right)}\right)}^{2},e^{\left(i\right)},K\right)$$

where ψ is a function that converts tracking error $e^{\left(i\right)}$ to an equivalent sensory noise and K is the stiffness of the elastic spring. This function changes with respect to the process noise $\sigma_{\mu }^{2}$ as the performance of the linear quadratic estimator is directly related to this value, and the controller strength q that affects how closely one can follow the estimated target trajectory.

To determine ψ, we simulated only the solo trials of the tracking task for each unique pair of $\sigma_{\mu }^{2}$ and q, and fitted a second order polynomial that related the standard deviation of an individual’s visual tracking noise $\sigma^{\left(i\right)}$ and tracking error $e^{\left(i\right)}$,

(18) $$\sigma^{\left(i\right)}=\gamma_{0}\;+\;\gamma_{1}\;e^{\left(i\right)}\;+\;\gamma_{2}(e^{\left(i\right)}{)}^{2}$$

where $\gamma_{0}$, $\gamma_{1}$ and $\gamma_{2}$ are fitted parameters. Since we assume that the softness of the interaction results in additive sensory noise, the haptic noise of Equation 16 is modified to

(19) $${\left(\sigma_{h}^{\left(i\right)}\right)}^{2}={\left(\gamma_{0}+\gamma_{1}\left[{\bar{e}}^{\left(i\right)}+E\left(K\right)\right]+\gamma_{2}{\left[{\bar{e}}^{\left(i\right)}+E\left(K\right)\right]}^{2}\right)}^{2}$$

where ${\bar{e}}^{\left(i\right)}$ is the average of the partners’ tracking error and $E\left(K\right)$ is the additional error from the interaction stiffness K, whose values were taken from the haptic tracking experiment.

To remove the effects of the additional noise due to the elastic coupling on the predicted performance improvement (as described in the Discussion), the compliance noise in Equation 16 was set to ${\left(\sigma_{c}^{\left(i\right)}\right)}^{2}=0$.

## Additional files

Transparent reporting form

### Data availability

All data generated or analysed during this study are included in the manuscript and supporting files.

## Appendix 1

### Sensitivity analysis

10.7554/eLife.41328.013 For each proposed model, we conducted a sensitivity analysis to compare their predictive power over a parameter space. As the length of the trial is sufficiently long and the tracking task is continuous, we used an infinite-horizon optimal controller with quadratic cost. Two parameters of the model are adjustable; $\sigma_{\mu }^{2}$, a multiplier for the Gaussian noise $\mu_{k}$ in the velocity, and q>0, a multiplier for the state cost Q in the controller. The control gain L in Equation 5 will minimize the cost functional

(A1) $$J\equiv \sum_{k=1}^{\infty }x_{k}^{T}\left(qQ\right)x_{k}+u_{k}^{T}Ru_{k}\;\;\;\;$$

where $x_{k}$ is the combined state vector, $u_{k}$ is the motor command, the state cost qQ is positive semi-definite and control cost R>0. $\sigma_{\mu }^{2}$ is a scaling factor of the process noise that determines the frequency content of a simulated individual’s movement. *q* modulates the strength of the individual controllers. $\sigma_{\mu }^{2}$ was bound within a range such that the trajectories were not jerky, and *q* was varied within a stable controllable range. These parameters were kept the same for all individuals in a collective. The sum of squared error (SSE) between the fit from the data and from the simulation was used as a metric for predictive power. The *neuromechanical goal sharing* model exhibits less error than the *no exchange* model at the parameters best fitting the experimental data (Figure 4—figure supplement 2). The *no exchange* model appears to fit the data best for large $\sigma_{\mu }^{2}$ where trajectories were less smooth. However, it always yielded linear, first order improvement curves as a function of the partners’ relative error, which was different from the data that exhibited second and third order components. Thus, the *neuromechanical goal sharing* model that explained both the curvature of the improvement curve and the effect of the collective’s size on the improvement best explained the empirical data.

## References

[bib1] Bahrami B, Olsen K, Latham PE, Roepstorff A, Rees G, Frith CD (2010). Optimally interacting minds. Science.

[bib2] Basdogan C, Ho C-H, Srinivasan MA, Slater M (2000). An experimental study on the role of touch in shared virtual environments. ACM Transactions on Computer-Human Interaction.

[bib3] Burdet E, Franklin DW, Milner TE (2013). Human Robotics: Neuromechanics and Motor Control.

[bib4] Ganesh G, Takagi A, Osu R, Yoshioka T, Kawato M, Burdet E (2014). Two is better than one: physical interactions improve motor performance in humans. Scientific Reports.

[bib5] Hastie R, Kameda T (2005). The robust beauty of majority rules in group decisions. Psychological Review.

[bib6] Hawley ML, Litovsky RY, Culling JF (2004). The benefit of binaural hearing in a cocktail party: effect of location and type of interferer. The Journal of the Acoustical Society of America.

[bib7] Kalman RE (1960). A new approach to linear filtering and prediction problems. Journal of Basic Engineering.

[bib8] Körding KP, Wolpert DM (2004). Bayesian integration in sensorimotor learning. Nature.

[bib9] Ljung L (1979). Asymptotic behavior of the extended Kalman filter as a parameter estimator for linear systems. IEEE Transactions on Automatic Control.

[bib10] Malysz P, Sirouspour S (2013). Task performance evaluation of asymmetric semiautonomous teleoperation of mobile twin-arm robotic manipulators. IEEE Transactions on Haptics.

[bib11] Parise CV, Ernst MO (2016). Correlation detection as a general mechanism for multisensory integration. Nature Communications.

[bib12] Reed KB, Peshkin MA (2008). Physical collaboration of Human-Human and Human-Robot teams. IEEE Transactions on Haptics.

[bib13] Sebanz N, Bekkering H, Knoblich G (2006). Joint action: bodies and minds moving together. Trends in Cognitive Sciences.

[bib14] Takagi A, Beckers N, Burdet E (2016). Motion plan changes predictably in dyadic reaching. PLOS ONE.

[bib15] Takagi A, Ganesh G, Yoshioka T, Kawato M, Burdet E (2017). Physically interacting individuals estimate the partner’s goal to enhance their movements. Nature Human Behaviour.

[bib16] Takagi A, Usai F, Ganesh G, Sanguineti V, Burdet E (2018). Haptic communication between humans is tuned by the hard or soft mechanics of interaction. PLOS Computational Biology.

[bib17] van der Wel RP, Knoblich G, Sebanz N (2011). Let the force be with Us: dyads exploit haptic coupling for coordination. Journal of Experimental Psychology: Human Perception and Performance.

[bib18] Webb NM (1991). Task-Related Verbal Interaction and Mathematics Learning in Small Groups. Journal for Research in Mathematics Education.

[bib19] Zhang C, Liu J (2015). On crowdsourced interactive live streaming: a Twitch.Tv-based measurement study. Arxiv.

