# Supplementary material for: Individuals physically interacting in a group rapidly coordinate their movement by estimating the collective goal
Source: eLife. 2019 Feb 12;8:e41328. doi: 10.7554/eLife.41328 (PMC6372281; doi:10.7554/eLife.41328)
Supplement: Peer review history

# Decision letter

**DOI:** 10.7554/eLife.41328.015

**Affiliations:** University of Western OntarioCanada; University of Western OntarioCanada; University of TübingenGermany

In the interests of transparency, eLife includes the editorial decision letter and accompanying author responses. A lightly edited version of the letter sent to the authors after peer review is shown, indicating the most substantive concerns; minor comments are not usually included.

Thank you for submitting your article "Individuals physically interacting in a group rapidly coordinate their movement by estimating the collective goal" for consideration by *eLife*. Your article has been reviewed by three peer reviewers, including Jörn Diedrichsen as the Reviewing Editor and Reviewer #1, and the evaluation has been overseen by Richard Ivry as the Senior Editor. The following individual involved in review of your submission has agreed to reveal their identity: Martin A Giese (Reviewer #2).

The reviewers have discussed the reviews with one another and the Reviewing Editor has drafted this decision to help you prepare a revised submission.

Summary:

The manuscript describes an experiment that studies the haptic interaction between groups of different size within a tracking task, where multiple agents manipulate together a goal point that is elastically coupled to the individual agents. The study investigates how tracking accuracy depends on the performance of the individuals and finds that performance is mainly determined by the best members of the group, instead of representing the average. A detailed quantitative analysis is provided, how the performance error relative to the average performance changes as function of the number of group members. In addition, three quantitative control models are compared with the data, finding that a model that assumes that control is determined by an estimate of the average target position provides the best fits of the data.

Essential revisions:

1) All reviewers agreed that the manuscript could profit from a better explanation/motivation of the different models. Specifically, the neuromechanical goal sharing model should be introduced alongside the other models, as it is the most intuitive expectation of how the results should come out, based on what is known from interacting dyads. Reviewer 1 found the other two models not very informative, writing "Given past work, the dyads model would seem to the main hypothesis to be tested. The two alternative hypotheses – the no exchange model and the source separation model – are not well motivated. The no-exchange model has been already rejected in the context of the dyadic interaction. So why should participants suddenly switch to being confused? Just because they know that they track together with multiple partners? The "neuromechanical goal sharing" model would be the logical prediction from what has been found in dyads already. The null-model spelled out in the third paragraph of the Introduction seems therefore somewhat contrived – and the real null model is exactly the "neuromechnical goal sharing". Similarly, the source separation model appears contrived in that the participants simply have no information on which to base the source separation on. It would have been very surprising if they could have done this, but the finding that they simply treat the experienced forces as if these come from one individual is the expected finding."

However, the other two reviewers saw clearly value in retaining them, but also pointed out that they need to be motivated better so we recommend that you keep all three but revise the paper to provide better motivation.

2) One key result is that individuals benefit from groups of 3 and 4 more than they do from groups of 2. The nature of this benefit needs to be more fully understood. An important confounding factor is the virtual stiffness between the hand of the subject and the group-averaged position of all the other hands (Discussion paragraph three). Can this factor fully explain the group effect? The reviewers thought answering this question may demand some new data, where the group size is varied while stiffness kept constant.

It appears that the performance might strongly dependent on learning, maybe also on learning processes that are specific for the different numbers of group members, or for the presence of specific individuals in the groups. Authors should discuss clearly if/how they control for differences in such learning processes.

# Reply: Author response

**DOI:** 10.7554/eLife.41328.016

Essential revisions:

1) All reviewers agreed that the manuscript could profit from a better explanation/motivation of the different models. Specifically, the neuromechanical goal sharing model should be introduced alongside the other models, as it is the most intuitive expectation of how the results should come out, based on what is known from interacting dyads. Reviewer 1 found the other two models not very informative, writing "Given past work, the dyads model would seem to the main hypothesis to be tested. The two alternative hypotheses – the no exchange model and the source separation model – are not well motivated. The no-exchange model has been already rejected in the context of the dyadic interaction. So why should participants suddenly switch to being confused? Just because they know that they track together with multiple partners? The "neuro mechanical goal sharing" model would be the logical prediction from what has been found in dyads already. The null-model spelled out in the third paragraph of the Introduction seems therefore somewhat contrived – and the real null model is exactly the "neuromechnical goal sharing". Similarly, the source separation model appears contrived in that the participants simply have no information on which to base the source separation on. It would have been very surprising if they could have done this, but the finding that they simply treat the experienced forces as if these come from one individual is the expected finding."

However, the other two reviewers saw clearly value in retaining them, but also pointed out that they need to be motivated better so we recommend that you keep all three but revise the paper to provide better motivation.

We agree with the reviewers that these models should be better motivated in the manuscript. According to our paper (Takagi et al., 2017), the two individuals of a dyad combine the haptic information from the interaction with the partner with their own visual information in a stochastically optimal manner, based on an internal representation of the partner’s target as a function of their state, that they form during the interaction. The beneficial effects of the coordination mechanism could be outweighed by the interaction dynamics from a connection to multiple partners, which may help inferior partners but will likely hinder superior ones. As such, it was unclear whether the improvements from the coordination mechanism hold during collective interaction, or whether the connection dynamics to a larger inertia system begin to outweigh these benefits. This motivates a comparison between the data and the predictions from the no exchange model (considering only the effect of the connection dynamics) alongside the neuromechanical goal sharing model. We have modified the Introduction’s last two paragraphs to motivate the use of these two models correspondingly.

On the other hand, the source separation model is merely a limiting case of haptics useful to elucidate the interaction mechanism. It is now presented only the Discussion (paragraph six) in order to show that maximal information transfer cannot occur through haptics, highlighting the physical limit of haptics.

2) One key result is that individuals benefit from groups of 3 and 4 more than they do from groups of 2. The nature of this benefit needs to be more fully understood. An important confounding factor is the virtual stiffness between the hand of the subject and the group-averaged position of all the other hands (Discussion paragraph three). Can this factor fully explain the group effect? The reviewers thought answering this question may demand some new data, where the group size is varied while stiffness kept constant.

Thank you for this feedback that prompted us to clarify the mechanism of haptic communication in a collective. We have rewritten the Discussion to elucidate this mechanism, integrating new simulations to clarify how the partners exchange haptic information and how the interaction mechanics contribute to the collective size effect. In the model, an individual integrates the haptic estimate of the collective target by relating the interaction force and the target position through a representation of the partner. In the previous studies (Takagi et al., 2017, 2018), the noise in the haptic measurement of the partner’s target from the interaction force was the partner’s visual tracking noise. In collective interaction, the interaction force is equivalent to being connected to the average of the partners’ hand position by a stiffer spring (as shown in Equation 6). Assuming stochastically independent partners’ visual tracking, the variance in the interaction force, and subsequently the collective target estimated from this force, is inversely proportional to the squared number of partners (see Equation 10 and 11). Thus, the haptic noise on the collective target is different from averaging the partners’ visual tracking noise, and will yield different predictions depending on the group size. This is illustrated in the simulation of Figures 5C and 5D, where we modified the neuromechanical goal sharing model such that the haptic noise on the collective target was equal to the partners’ average noise (we also removed the dynamics and the compliance noise). This modified model yields similar improvements for dyads, triads and tetrads. Thus, the reduction in the variance in the interaction force due to the averaging of the noisy partners’ positions explains why the improvement is larger in bigger groups even if the partners’ average error is equivalent.

It appears that the performance might strongly dependent on learning, maybe also on learning processes that are specific for the different numbers of group members, or for the presence of specific individuals in the groups. Authors should discuss clearly if/how they control for differences in such learning processes.

We examined the deviation from the final improvement (shown in Figure 3D) for all individuals in every twos and fours recruited in our study as illustrated in Author response image 1. The rate at which individuals reached their final improvement was highly correlated between individuals in the same group (ρ=0.995 ± 0.001 for dyads and ρ=0.996 ± 0.0009 for tetrads), making it difficult to assess individual contributions to the improvement observed in a particular dyad or tetrad. The performance improvement of the individuals in a particular group are correlated likely due to the dynamics. As such, the improvements from a group should be treated as a random factor that can modulate the group’s performance improvement. This factor is controlled for in the linear-mixed effects analysis of the performance improvement, by assuming that each recruited group of two and fours is a random factor that influences the performance improvement (refer to Equation 2 in the Materials and methods). This is now discussed in the Results when describing the linear mixed-effects analysis of the improvement curves in Figure 3B.

**Author response image 1.** Deviation from the final improvement as a function of the time during the trial for every individual (denoted by a different colour) in all twos (left panels) and fours (right panels) recruited in our study. The rates of convergence to the final improvement differs somewhat between different groups, but it was highly correlated between individuals of the same group. As such, it is difficult to isolate the contribution of a specific individual to the improvement of a collective, and so each group of twos and fours must be treated as a random factor in the analysis.
